# *Dentipellistasmanica* sp. nov. (Hericiaceae, Basidiomycota) from Australia

**DOI:** 10.3897/mycokeys.41.28485

**Published:** 2018-10-11

**Authors:** Xiao-Hong Ji, Qian Chen, Genevieve Gates, Ping Du

**Affiliations:** 1 Institute of Microbiology, PO Box 61, Beijing Forestry University, Beijing 100083, China Beijing Forestry University Beijing China; 2 Tasmanian Institute of Agriculture, Private Bag 54, Hobart, Tasmania 7001, Australia Tasmanian Institute of Agriculture Tasmania Australia; 3 College of Life Science and Technology, Yangtze Normal University, Chongqing 408100, China Yangtze Normal University Chongqing China

**Keywords:** hydnoid fungi, *
Russulales
*, taxonomy, wood-inhabiting fungi

## Abstract

*Dentipellistasmanica***sp. nov.** is described and illustrated from Tasmania, Australia based on rDNA evidence and morphological characters. It is characterised by an annual growth habit; resupinate basidiocarps up to 100 cm long; spines cream when fresh and cinnamon when dry, up to 3 mm long and a few glued at tips when dry; distinct white fibrillous to cottony margin; a monomitic hyphal structure with non-amyloid, non-dextrinoid and cyanophilous generative hyphae; the presence of gloeoplerous hyphae and gloeocystidia which become dark blue in Melzer’s reagent; the presence of chlamydospores in the subiculum and rough basidiospores measuring 3.5–4.5 × 2.4–3.2 µm. A molecular study based on the combined ITS (internal transcribed spacer region) and 28S (the large nuclear ribosomal RNA subunit) dataset supports the new species in *Dentipellis*. A key to species of *Dentipellis* sensu stricto is provided.

## Introduction

*Dentipellis* Donk, typified by *D.fragilis* (Pers.) Donk, is a hydnaceous genus in the Russulales and is characterised by a wood-inhabiting resupinate fruiting body with soft spines, a monomitic hyphal structure with clamp connections on the generative hyphae and amyloid, rough basidiospores (Ginns 1986, Dai et al. 2009, Zhou and Dai 2013). Zhou and Dai (2013) demonstrated that *Dentipellis* was polyphyletic and segregated *Dentipellisleptodon* (Mont.) Maas Geest. and *Dentipellistaiwaniana* Sheng H. Wu from *Dentipellis* to a new genus of *Dentipellicula* Y.C. Dai & L.W. Zhou based on ITS and 28S rDNA sequences. Besides, *Dentipellopsis* Y.C. Dai & L.W. Zhou is erected as a new genus and characters are provided in a generic key to distinguish *Dentipellicula*, *Dentipellis* and *Dentipellopsis* that morphologically are highly similar, as well as a key to the current species in *Dentipellis* (Zhou and Dai 2013). Recently, based on molecular and morphological analyses, more new taxa were described in *Dentipellis* sensu lato (Zhou and Dai 2013, Chen et al. 2015, Shen and Wang 2017, Yuan et al. 2018) and, indeed, all *Dentipellis* spp. were found from the northern Hemisphere (Ginns 1986, Dai et al. 2009, Zhou and Dai 2013, Shen and Wang 2017, Yuan et al. 2018).

During a field trip to Tasmania, the island state of Australia, three wood-inhabiting specimens with soft spines were collected and, based on the morphological characters, they belong to *Dentipellis*. After phylogenetic analysis of ITS and 28S sequences and examination of the morphology in the laboratory, they turn out to represent a new species. This is so far the first species of *Dentipellis* found in the southern Hemisphere. In this paper, we present an illustrated description and an identification key to accepted species of *Dentipellis* worldwide.

## Materials and methods

### Morphological studies

Thin sections were studied microscopically according to Chen et al. (2016) at magnifications ≤1000× using a Nikon Eclipse 80i microscope with phase contrast illumination. Drawings were made with the aid of a drawing tube. Microscopic features, measurements and drawings were made from sections stained with Cotton Blue and Melzer’s reagent. Spores were measured from sections cut from the tubes. To present spore size variation, the 5% of measurements excluded from each end of the range are given in parentheses. Basidiospore apiculus lengths were not included in the measurements.

Abbreviations include:

IKI Melzer’s reagent,

IKI– negative in Melzer’s reagent,

IKI+ amyloid in Melzer’s reagent,

KOH 5% potassium hydroxide,

CB Cotton Blue,

CB+ cyanophilous,

CB– acyanophilous,

L mean spore length (arithmetic average of all spores),

W mean spore width (arithmetic average of all spores),

Q the L/W ratio,

n number of spores measured from the given number of specimens.

Colour terms follow Petersen (1996). The studied specimens are deposited in the herbaria as cited below; herbarium abbreviations follow Thiers (2014).

### Molecular study and phylogenetic analysis

A CTAB rapid plant genome extraction kit (Aidlab Biotechnologies, Beijing) was used to obtain PCR products from dried specimens, according to the manufacturer’s instructions with some modifications (Wu et al. 2017). The primer pair ITS4 and ITS5 was used for amplification of the ITS region (White et al. 1990), while the primer pair LR0R and LR7 (http://www.biology.duke.edu/fungi/mycolab/primers.htm) was used for providing the D1-D4 regions of the 28S (https://unite.ut.ee/primers.php). The PCR procedure for ITS was as follows: initial denaturation at 95 °C for 3 min, followed by 35 cycles at 94 °C for 40 s, 54 °C for 45 s and 72 °C for 1 min, with a final extension of 72 °C for 10 min. The PCR procedure for 28S was as follows: initial denaturation at 94 °C for 1 min, followed by 35 cycles at 94 °C for 30 s, 50 °C for 1 min and 72 °C for 1.5 min, with a final extension of 72 °C for 10 min. The PCR products were purified and sequenced in Beijing Genomics Institute, China with the same primers.

New sequences, deposited in GenBank (Table 1), were aligned with additional sequences retrieved from GenBank (Table 1) using BioEdit 7.0.5.3 (Hall 1999) and ClustalX 1.83 (Chenna et al. 2003). *Bondarzewiapodocarpi* Y.C. Dai & B.K. Cui and *B.occidentalis* Jia J. Chen, B.K. Cui & Y.C. Dai were chosen as outgroups, consulting Dai et al. (2010) and Zhou and Dai (2013). Prior to phylogenetic analysis, ambiguous regions at the start and the end of the alignment were deleted and gaps were manually adjusted to optimise the alignment. The edited alignment was deposited at TreeBase (submission ID 22975; www.treebase.org).

**Table 1.** Specimens and GenBank accession number of sequences used in this study.

Species	Sample no.	Locality	GenBank accession no.	
			ITS	nLSU
* Bondarzewia occidentalis *	DAOM F-415	Canada	DQ200923	DQ234539
* B. podocarpi *	Dai 9261	China	KJ583207	KJ583221
* Dentipellicula austroafricana *	Dai 12580	South Africa	KJ855274	KJ855275
* D. leptodon *	GB 011123	Uganda	EU118625	EU118625
* D. taiwaniana *	Dai 10867	China	JQ349115	JQ349101
	Cui 8346	China	JQ349114	JQ349100
* Dentipellis coniferarum *	Cui 10063	China	JQ349106	JQ349092
	Yuan 5623	China	JQ349107	JQ349093
* D. dissita *	NH 6280	Canada	AF506386	AF506386
* D. fragilis *	Dai 12550	China	JQ349110	JQ349096
	Dai 9009	China	JQ349108	JQ349094
* D. longiuscula *	He 20120717-5	China	KR108235	KR108238
	He 20120717-7	China	KR108234	KR108239
* D. microspora *	Cui 10035	China	JQ349112	JQ349098
* D. rhizomorpha *	Dai 17474	China	MG020134	MG020137
	Dai 17477	China	MG020135	MG020138
	Dai 17481	China	MG020136	MG020139
*** D. tasmanica ***	**Dai 18737**	**China**	**MH571698^a^**	**MH571701^a^**
	**Dai 18767**	**China**	**MH571699^a^**	**MH571702^a^**
	**Dai 18768**	**China**	**MH571700^a^**	**MH571703^a^**
* D. tropicalis *	Cui 8545	China	KR108236	KR108240
	He 1993	China	KR108237	KR108241
* Dentipellopsis dacrydicola *	Dai 12004	China	JQ349104	JQ349089
* D. dacrydicola *	Dai 12010	China	–	JQ349090
* Hericium abietis *	NH 6990	Canada	AF506456	AF506456
* H. alpestre *	NH 13240	Russia	AF506457	AF506457
* H. americanum *	DAOM F-21467	Canada	AF506458	AF506458
* H. coralloides *	NH 282	Sweden	AF506459	AF506459
* H. erinaceus *	NH 12163	Russia	AF506460	AF506460
* Laxitextum bicolor *	NH 5166	Sweden	AF310102	AF310102
* Pseudowrightoporia japonica *	Dai 7221	China	FJ644289	KM107882
* Wrightoporiopsis biennis *	Cui 8457	China	KJ807066	KJ807074

^a^ Sequences newly generated in this study; the new species is shown in bold.

The method of phylogenetic analysis followed Chen et al. (2016). Maximum parsimony (MP) analysis was performed in PAUP* version 4.0b10 (Swofford 2002). All characters were equally weighted and gaps were treated as missing data. Trees were inferred using the heuristic search option with tree-bisection reconnection (TBR) branch swapping and 1,000 random sequence additions. Max-trees were set to 5,000, branches of zero length were collapsed and all parsimonious trees were saved. Clade robustness was assessed using a bootstrap (BT) analysis with 1,000 replicates (Felsenstein 1985). Phylogenetic trees were visualised using Treeview (Page 1996).

MrModeltest 2.3 (Posada and Crandall 1998, Nylander 2004) was used to determine the best-fit evolution model of the combined dataset for Bayesian Inference (BI). BI was calculated with MrBayes 3.1.2 (Ronquist and Huelsenbeck 2003) with a general time reversible (GTR) model of DNA substitution and an invgamma distribution rate variation across sites. Four Markov chains were performed for 2 runs from random starting trees for 500,000 generations of the combined ITS and 28S dataset and trees were sampled every 100 generations. The burn-in was set to discard the first 25% of the trees. A majority rule consensus tree of all remaining trees was calculated. Nodes that received BT support ≥50% and Bayesian posterior probabilities (BPP) ≥0.95 were considered as significantly supported.

## Results

**Figure 1.**
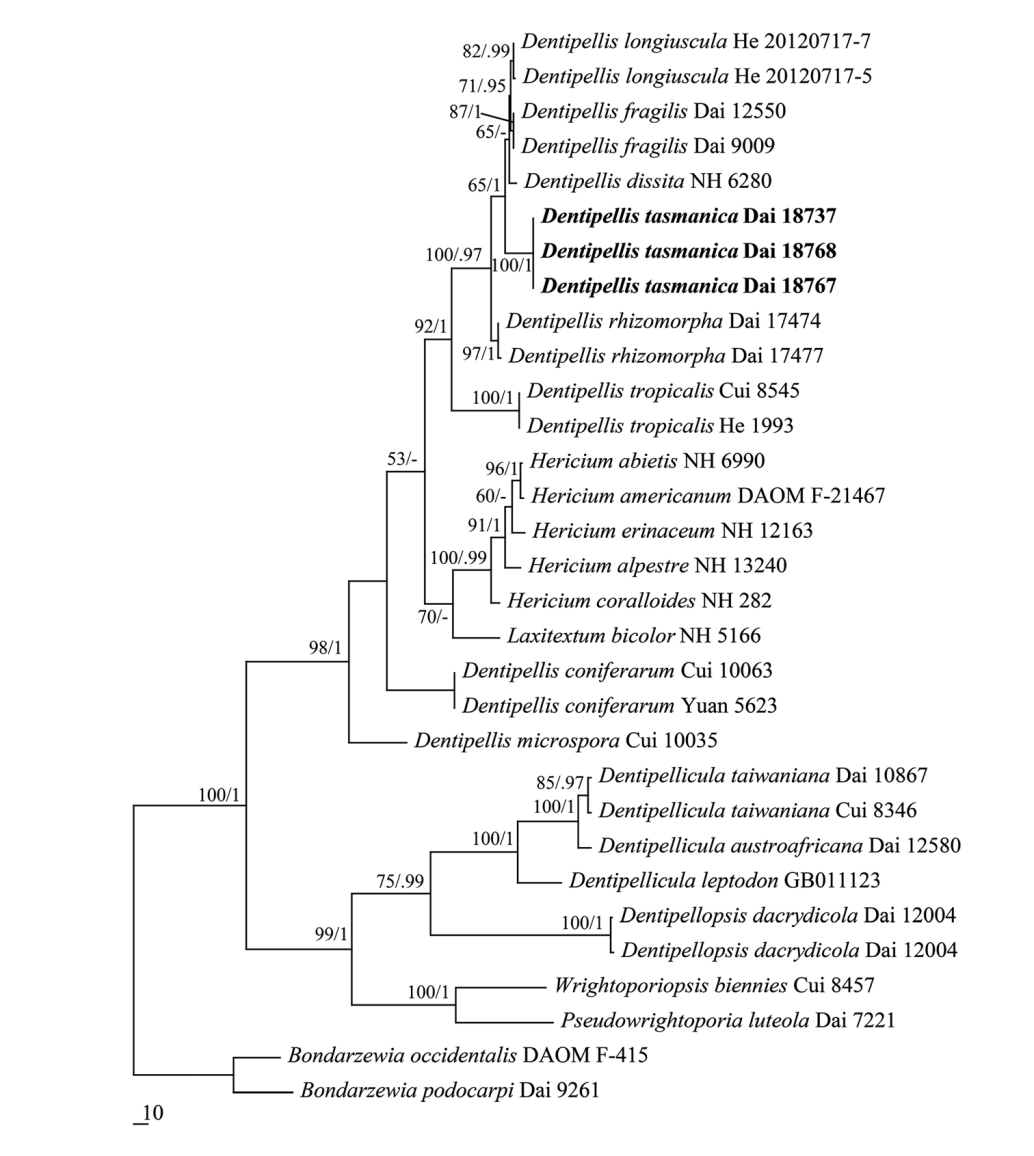
Strict consensus tree illustrating the phylogenetic position of *Dentipellistasmanica*, generated by the maximum parsimony method based on ITS+28S sequence data. Branches are labelled with parsimony bootstrap values ≥50% and Bayesian posterior probabilities ≥0.95. *Bondarzewiapodocarpi* and *B.occidentalis* are used to root the tree. Branch lengths reflect expected changes per site as indicated by the scale.

The combined ITS and 28S dataset included sequences from 31 fungal collections representing 22 species. The dataset had an aligned length of 1792 characters, of which 1218 characters are constant, 126 are variable and parsimony-uninformative and 448 (37%) are parsimony-informative. MP analysis yielded 2 equally parsimonious trees (TL = 1343, CI = 0. 653, RI = 0.793, RC = 0.518, HI = 0.347). The best-fit model for the combined ITS and 28S sequences dataset estimated and applied in the Bayesian analysis: GTR+I+G, lset nst = 6, rates = invgamma; prset statefreqpr = dirichlet (1,1,1,1). BIresulted in a similar topology with an average standard deviation of split frequencies = 0.006203 to MP analysis and, thus, only the MP tree was provided. Both BT values (≥50%) and BPPs (≥0.95) are shown at the nodes (Fig. 1).

Three sampled specimens of the new species, *Dentipellistasmanica*, formed a well-supported lineage (100% MP and 1 BPPs), indicating they are phylogenetically distinct from other species (Fig. 1).

## Taxonomy

### Dentipellis tasmanica

Taxon classificationFungiRussulalesHericiaceae

Y.C. Dai, G.M. Gates, X.H. Ji & P. Du sp. nov.

MB827073

Figs 1 , 2

#### Diagnosis.

Differs from other *Dentipellis* species by its gloeoplerous hyphae and gloeocystidia that become dark blue in Melzer’s reagent and the presence of chlamydospores in subiculum.

#### Holotype.

AUSTRALIA. Tasmania: Arve River Streamside Reserve, 43°10'S, 146°48.5'E, elev. 160 m, on fallen trunk of *Nothofagus* sp., 15 May 2018, *Dai 18767* (M, isotype in BJFC; ITS GenBank accession number: MH571699, 28S GenBank accession number: MH571702).

#### Etymology.

*Tasmanica* (Lat.): referring to the species collected from Tasmania of Australia.

#### Basidiomata.

Annual, resupinate, inseparable from substratum, soft corky, without odour or taste when fresh, fragile upon drying, up to 100 cm long, 40 cm wide and 3.5 mm thick at centre. Hymenophore with spines, cream when fresh and cinnamon when dry, spines up to 3 mm long, 2–3 per mm across base, soft corky to fragile, a few glued at tips when dry; margin distinct, white, fibrillous to cottony, up to 5 mm wide; spines, cream, becoming fragile and clay-buff when dry, up to 3 mm long. Subiculum very thin, soft corky, white to cream, <1 mm thick.

#### Hyphal structure.

Hyphal system monomitic; generative hyphae with clamp connections, IKI–, CB+; the colour and size unchanged in KOH.

#### Subiculum.

Generative hyphae colourless, thin- to slightly thick-walled, frequently branched, flexuous, interwoven, 3–4.5 μm in diam. Gloeoplerous hyphae occasionally present, dark blue in Melzer’s reagent. Chlamydospores present, ellipsoid, thick-walled, 5–5.6 × 2.8–3.3 μm.

#### Hymenophoral trama.

Generative hyphae colourless, thin-walled, frequently branched, straight, parallel along the spines, 2.8–4 μm in diam. Gloeocystidia abundant, colourless, thin- to slightly thick-walled, clavate, contents oily and dark blue in Melzer’s reagent, rooting deep from the trama, up to a few hundred microns long, the cystidia-like apical part 30–45 × 5–8 μm. Oily material abundant amongst trama.

#### Hymenium.

Cystidioles colorless, thin-walled, ventricose with elongated apical portion, bearing some irregular crystals, 30–45 × 5–8 μm; basidia clavate with four sterigmata and a basal clamp connection, 20–26 × 3–4.5 μm. Basidiospores ellipsoid, coloruless, thin-walled, densely echinulate, IKI+, CB+, (3.4–)3.5–4.5(–4.8) × 2.4–3.2(–3.5) μm, L = 3.99 μm, W = 2.92 μm, Q = 1.36–1.39 (n = 90/3).

#### Additional specimens examined (paratypes).

AUSTRALIA. Tasmania: Arve River Streamside Reserve, on fallen trunk of *Nothofagus* sp., 15 May 2018, *Dai 18768* (M, duplicate in BJFC; ITS GenBank accession number: MH571700, 28S GenBank accession number: MH571703); Mt Field National Park, 42°41'S, 146°42'E, elev., 180 m, on fallen trunk of *Nothofagus* sp., 14 May 2018, *Dai 18737* (M, duplicate in BJFC; ITS GenBank accession number: MH571698, 28S GenBank accession number: MH571701).

**Figure 2.**
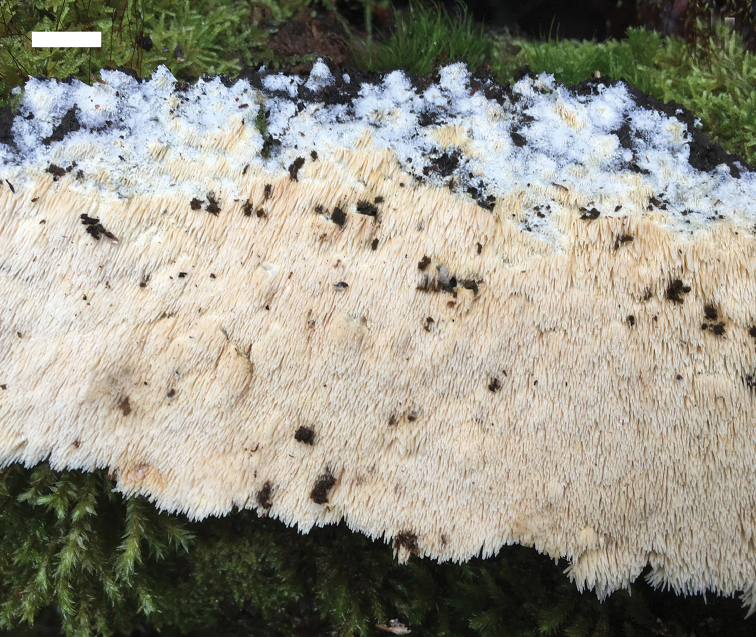
A fresh basidiocarp of *Dentipellistasmanica* (holotype). Scale bar: 1 cm.

**Figure 3.**
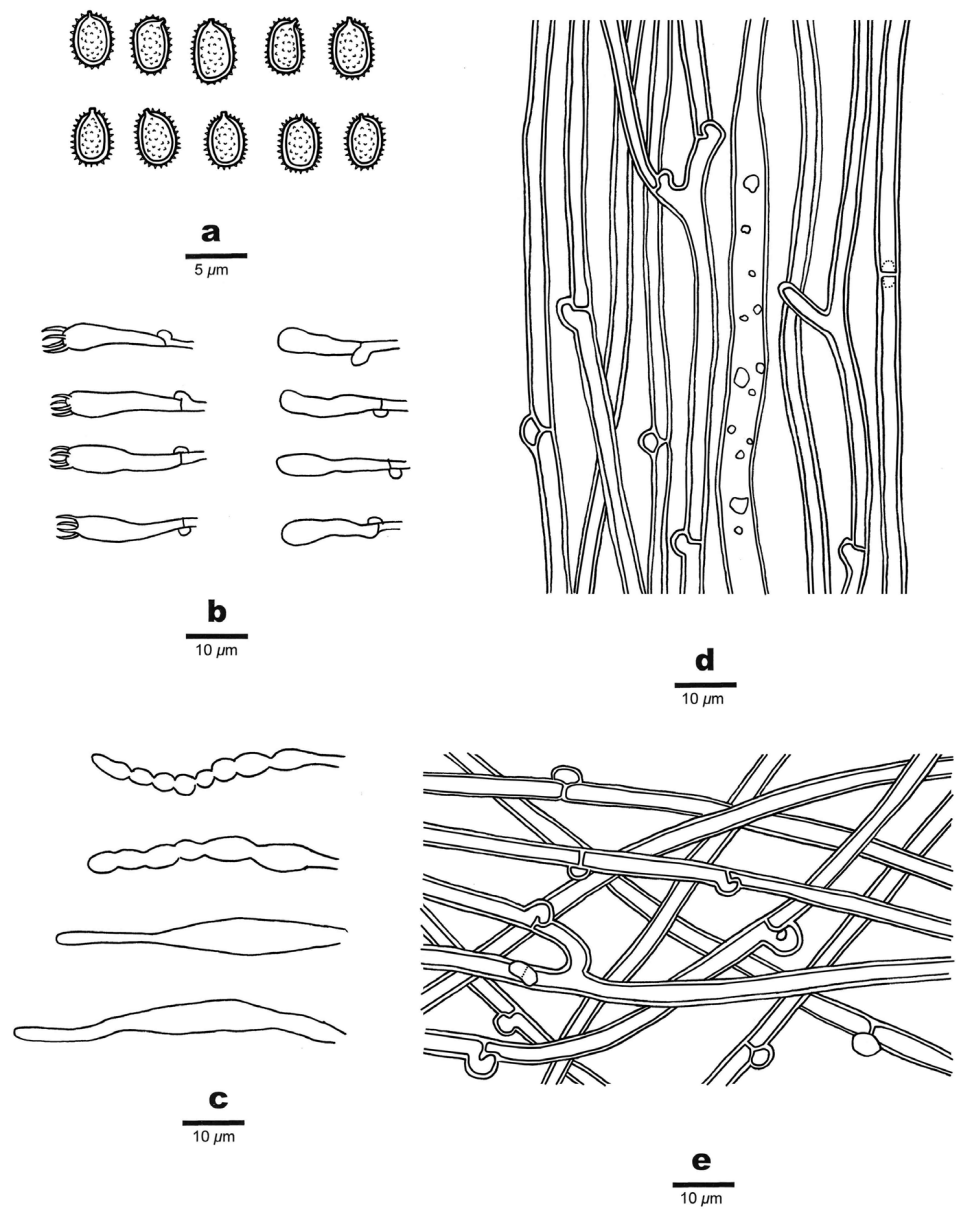
Microscopic structures of *Dentipellistasmanica* (holotype). **a** Basidiospores **b** Basidia and basidioles **c** Gloeocystidia and Cystidioles **d** Hyphae from trama **e** Hyphae from subiculum.

## Discussion

Morphologically, *Dentipellistasmanica* is characterised by spines, cream when fresh; distinct white fibrillous to cottony margin; a monomitic hyphal structure with generative hyphae bearing clamp connections; the presence of gloeoplerous hyphae and gloeocystidia which become dark blue in Melzer’s reagent and presence of chlamydospores in the subiculum. Phylogenetically, three samples of *D.tasmanica* formed a distinct lineage with strong support (100 % MP, 1.0 BPPs) and are distant from other taxa (Fig. 1). Both morphology and rDNA sequence data confirmed that *D.tasmanica* is a new species in *Dentipellis*.

*Dentipellistasmanica* was considered as *Dentipelliculaleptodon* (Mont.) Y.C. Dai & L.W. Zhou (Gates and Ratkowsky 2016) as having similar basidiospores (3.5–4.5 × 2.4–3.3 μm vs. 3.2–4 1 × 2.4–3 µm, Ginns 1986), but gloeocystidia and gloeoplerous hyphae in *D.leptodon* are yellowish in Melzer’s reagent and it lacks chlamydospores in subiculum.

Phylogenetically, *Dentipellistasmanica* is more closely related to *D.rhizomorpha* Yuan & Y.C. Dai, *D.fragilis*, *D.dissita* and *D.longiuscula* (Fig. 1). However, *D.rhizomorpha* has denser spines (5–7 per mm vs. 2–3 per mm in *D.tasmanica*), lacks gloeoplerous hyphae and gloeocystidia. *D.fragilis* and *D.dissita* differ from *D.tasmanica* in having larger basidiospores (5–5.8 × 4.1–4.9 μm in *D.fragilis*, 4.2–4.7 × 3.2–3.7 μm in *D.dissita*; Dai et al. 2009). *D.longiuscula* is distinguished from *D.tasmanica* by lacking gloeoplerous hyphae and gloeocystidia and having larger basidiospores (5–6 × 3–3.6 μm; Shen and Wang 2017).

### Key to species of *Dentipellis*

**Table:** 

1	Gloeoplerous hyphae absent	**2**
–	Gloeoplerous hyphae present	**5**
2	Basidiospores <5 μm long	**3**
–	Basidiospores ≥5 μm long	**4**
3	Basidiospores <3.2 µm long, <2.2 µm wide-	*** D. microspora ***
–	Basidiospores >3.2 µm long, >2.2 µm wide-	*** D. rhizomorpha ***
4	Gloeocystidia absent	*** D. longiuscula ***
–	Gloeocystidia present	*** D. tropicalis ***
5	Basidiocarps becoming brown when bruised	*** D. coniferarum ***
–	Basidiocarps unchanged when bruised	**6**
6	Gloeocystidia absent	*** D. ohiensis ***
–	Gloeocystidia present	**7**
7	Gloeocystidia dark blue in IKI, basidiospores <3.2 μm wide	*** D. tasmanica ***
–	Gloeocystidia yellowish in IKI, basidiospores >3.2 μm wide	**8**
8	Basidiospores 5–5.8 × 4.1–4.9 μm	*** D. fragilis ***
–	Basidiospores 4.2–4.7 × 3.2–3.7 μm	*** D. dissita ***

## Supplementary Material

XML Treatment for Dentipellis tasmanica

## References

[B1] Chen JJ Cui BK Dai YC (2016) Global diversity and molecular systematics of *Wrightoporia* s. l. (Russulales, Basidiomycota). Persoonia 37: 21–36. 10.3767/003158516X689666 28232759 PMC5315289

[B2] Chen JJ Cui BK He SH Cooper JA Barrett MD Chen JL Dai YC (2016) Molecular phylogeny and global diversity of the remarkable genus *Bondarzewia* (Basidiomycota, Russulales). Mycologia 108: 697–708. 10.3852/14-216 27091389

[B3] Chen JJ Shen LL Dai YC (2015) *Dentipelliculaaustroafricana* sp. nov. supported by morphological and phylogenetic analyses. Mycotaxon 130: 17–25. 10.5248/130.17

[B4] Chenna R Sugawara H Koike T Lopez R Gibson TJ Higgins DG Thompson JD (2003) Multiple sequence alignment with the Clustal series of programs. Nucleic Acids Research 31: 3497–3500. 10.1093/nar/gkg500 12824352 PMC168907

[B5] Dai YC Cui BK Liu XY (2010) *Bondarzewiapodocarpi*, a new and remarkable polypore from tropical China. Mycologia 102: 881–886. 10.3852/09-050 20648754

[B6] Dai YC Xiong HY Wu SH (2009) Notes on *Dentipellis* (Russulales, Basidiomycota). Mycosystema 28: 668–671.

[B7] Felsenstein J (1985) Confidence intervals on phylogenetics: an approach using bootstrap. Evolution 39: 783–791. 10.2307/2408678 28561359

[B8] Gates G Ratkowsky D (2016) A field guide to Tasmanian fungi. Tasmanian Field Naturalists Club, Hobart, 1–249.

[B9] Ginns J (1986) The genus *Dentipellis* (Hericiaceae). Windahlia 16: 35–45.

[B10] Hall TA (1999) Bioedit: a user-friendly biological sequence alignment editor and analysis program for Windows 95/98/NT. Nucleic Acids Symp Ser 41: 95–98

[B11] Nylander JAA (2004) MrModeltest v2. Program distributed by the author. Evolutionary Biology Centre, Uppsala University.

[B12] Page RMD (1996) Treeview: An application to display phylogenetic trees on personal computers. Comput Appl Biosci 12: 357–358. 8902363 10.1093/bioinformatics/12.4.357

[B13] Petersen JH (1996) The Danish Mycological Society´s color-chart. Foreningen til Svampekundskabens Fremme, Greve, 1–6.

[B14] Posada D Crandall KA (1998) Modeltest: testing the model of DNA substitution. Bioinformatics 14: 817–818. 10.1093/bioinformatics/14.9.817 9918953

[B15] Ronquist F Huelsenbeck JP (2003) MRBAYES 3: Bayesian phylogenetic inference under mixed models. Bioinformatics 19: 1572–1574. 10.1093/bioinformatics/btg180 12912839

[B16] Shen LL Wang M (2017) Morphological characteristics and molecular data reveal two new species of *Dentipellis* from China. Phytotaxa 323: 69. 10.11646/phytotaxa.323.1.5

[B17] Swofford DL (2002) PAUP*: Phylogenetic analysis using parsimony (*and other methods). Version 4.0b10. Sinauer Associates, Sunderland, Massachusetts.

[B18] Thiers B (2014) Index Herbariorum: A global directory of public herbaria and associated staff. New York Botanical Garden’s Virtual Herbarium.

[B19] White TJ Bruns TD Lee S Taylor J (1990) Amplification and direct sequencing of fungal ribosomal RNA genes for phylogenetics. In: Innis MA Gelfand DH Sninsky JJ White TJ (Eds) PCR protocols, a guide to methods and applications.Academic, San Diego, 315–322. 10.1016/B978-0-12-372180-8.50042-1

[B20] Wu F Chen JJ Ji XH Vlasák J Dai YC (2017) Phylogeny and diversity of the morphologically similar polypore genera *Rigidoporus, Physisporinus, Oxyporus* and *Leucophellinus*. Mycologia 109: 749–765. 10.1080/00275514.2017.1405215 29336678

[B21] Yuan Y Ren GJ Dai YC (2018) *Dentipellisrhizomorpha* sp. nov. supported by morphological and phylogenetic analyses. Nova Hedwigia 107: 131–140. 10.1127/nova_hedwigia/2018/0459

[B22] Zhou LW Dai YC (2013) Taxonomy and phylogeny of hydnoid Russulales: two new genera, three new species and two new combination species. Mycologia 105: 636–649. 10.3852/12-011 23360974

